# Evaluating noninvasive brain stimulation to treat overactive bladder in individuals with multiple sclerosis: a randomized controlled trial protocol

**DOI:** 10.1186/s12894-023-01358-8

**Published:** 2024-01-25

**Authors:** Betsy H. Salazar, Kristopher A. Hoffman, John A. Lincoln, Christof Karmonik, Hamida Rajab, Santosh A. Helekar, Rose Khavari

**Affiliations:** 1 Department of Urology, Houston Methodist Hospital, 6560 Fannin St. Suite 2100, Houston, TX 77030 USA; 2 Translational Imaging Center, Houston Methodist Research Institute, Houston, TX USA; 3 Department of Neurology, The University of Texas Health Science Center, Houston, TX USA; 4 Center for Translational Biomagnetics and Neurometry, Houston Methodist Research Institute, Houston, TX USA

**Keywords:** Multiple sclerosis, Neurogenic overactive bladder, Functional MRI, Transcranial magnetic stimulation

## Abstract

**Background:**

Multiple Sclerosis (MS) is an often debilitating disease affecting the myelin sheath that encompasses neurons. It can be accompanied by a myriad of pathologies and adverse effects such as neurogenic lower urinary tract dysfunction (NLUTD). Current treatment modalities for resolving NLUTD focus mainly on alleviating symptoms while the source of the discomfort emanates from a disruption in brain to bladder neural circuitry. Here, we leverage functional magnetic resonance imaging (fMRI), repetitive transcranial magnetic stimulation (rTMS) protocols and the brains innate neural plasticity to aid in resolving overactive bladder (OAB) symptoms associated with NLUTD.

**Methods:**

By employing an advanced neuro-navigation technique along with processed fMRI and diffusion tensor imaging data to help locate specific targets in each participant brain, we are able to deliver tailored neuromodulation protocols and affect either an excitatory (20 min @ 10 Hz, applied to the lateral and medial pre-frontal cortex) or inhibitory (20 min @ 1 Hz, applied to the pelvic supplemental motor area) signal on neural circuitry fundamental to the micturition cycle in humans to restore or reroute autonomic and sensorimotor activity between the brain and bladder. Through a regimen of questionnaires, bladder diaries, stimulation sessions and analysis, we aim to gauge rTMS effectiveness in women with clinically stable MS.

**Discussion:**

Some limitations do exist with this study. In targeting the MS population, the stochastic nature of MS in general highlights difficulties in recruiting enough participants with similar symptomology to make meaningful comparisons. As well, for this neuromodulatory approach to achieve some rate of success, there must be enough intact white matter in specific brain regions to receive effective stimulation. While we understand that our results will represent only a subset of the MS community, we are confident that we will accomplish our goal of increasing the quality of life for those burdened with MS and NLUTD.

**Trial registration:**

This trial is registered at ClinicalTrials.gov (NCT06072703), posted on Oct 10, 2023.

## Background

Multiple sclerosis (MS) is a disease characterized by the loss of myelin in multiple areas of the central nervous system. Neurogenic Overactive Bladder (NOAB) is a condition defined by the International Continence Society, which involves frequent urination, or a strong and urgent desire to urinate, with or without incontinence (UUI) [1, 2]. This condition affects approximately 88% of individuals with MS and is the most common urinary problem experienced by these patients [3, 4]. The current treatment options for NOAB in MS patients encompass a variety of approaches. Behavioral therapies such as bladder training, pelvic floor muscle training, and fluid management are commonly used as a first-line therapy [5–7]. Additionally, anticholinergics (ACs) with or without intermittent self-catheterization can be considered. However, the use of ACs carries risks of cognitive decline, memory degradation, constipation, and impaired bladder emptying, which are particularly concerning for individuals with MS [8–11]. Another treatment option is the intravesical injection of OnabotulinumtoxinA (BTX-A), which has proven effective in managing NOAB. However, its impact on the MS population tends to be moderate, with only 43% of patients achieving complete dryness after treatment [12]. Furthermore, up to 56% of patients experience urinary tract infections, which can worsen urinary and neurological symptoms [13]. More invasive approaches for managing NOAB in MS patients involve neurostimulation techniques such as percutaneous tibial nerve stimulation (PTNS) [14] and sacral neuromodulation (SNS), however, these methods remain experimental with modest results and some adverse side effects [15–19].

To overcome the limitations of traditional treatments, we shift our focus from symptom management symptoms to treating NOAB by utilizing repetitive Transcranial Magnetic Stimulation (rTMS), which leverages the brain's regulatory role in bladder function. rTMS is a safe and noninvasive technique that involves placing an electromagnetic coil near the scalp to deliver pulsed magnetic fields to the cortex, thereby modulating neurons without the need for anesthesia or causing significant side effects [20, 21], This technique has been widely used for brain mapping, treating drug-resistant depression, migraines, and obsessive–compulsive disorders [22–24]. Previous small-scale studies (without a placebo group) have demonstrated the feasibility and effectiveness of rTMS targeted at inhibiting the supplementary motor area (SMA) in individuals with MS and Parkinson's disease, suggesting its potential to improve NLUTD [25, 26].

**Fig. 1:**
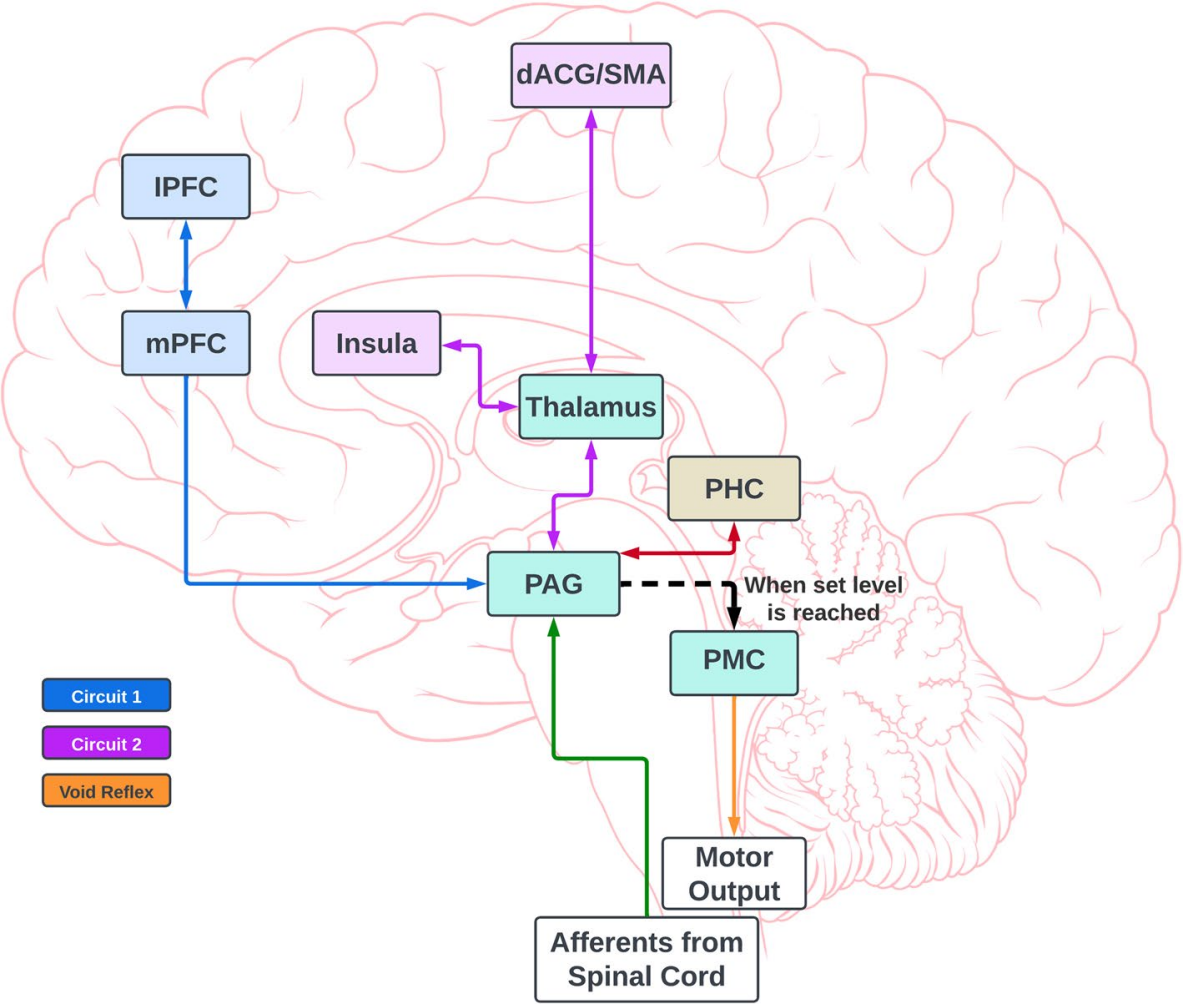
Brain-bladder circuitry

The challenge of specifically targeting the brain regions responsible for controlling the micturition cycle has been thoroughly investigated in parallel with developments in functional magnetic resonance imaging (fMRI) technology [27]. Through fMRI, researchers have discovered distinct neural circuits involved in regulating the lower urinary tract (LUT), and developed a working model of communication between the brain and bladder (Fig. 1).

Numerous researchers have demonstrated that the patterns of activation within these circuits are modified in people experiencing symptoms of OAB [28, 29]. Our research team has extensively examined how the brain controls LUT functions in women with MS [30–40]. We have also observed similar results indicating elevated BOLD signals in these specific ROIs within circuit 2 when individuals with MS and NOAB experience a strong urge to void, suggesting an abnormal perception of bladder volume in these individuals [32, 35]. Although the precise mechanisms governing the interactions among these circuits are not yet understood, it is now apparent that they do not operate independently in either healthy individuals or those with OAB/NOAB [29]. Recent data support the notion that the activation patterns of these networks are associated with the amount of urine present in the bladder and its corresponding response, particularly the sensation of urgency [41]. Based on this, we proposed that alterations in cortical bladder volume perception contribute to symptoms in MS patients with NOAB, and that by enhancing the response of circuits 1 and 2 to bladder distention through neuronavigated rTMS applied to two specific ROIs involved in LUT, we can restore brain activity and alleviate symptoms (such as frequency, urgency, and incontinence) when compared to a sham rTMS procedure.

## Methods and design

### Study design

This is a phase II randomized, double-blind, sham-controlled clinical trial with an optional open-label extension (OLE) phase aimed at assessing the effects of targeted rTMS in women with MS and NOAB. The trial aims to investigate the restoration of brain function and improvement in urinary frequency, urgency, and incontinence. Participants undergo a minimum of 10 rTMS treatment sessions, with the option to continue in the OLE phase. Clinical and neuroimaging data are collected at baseline and post-treatment to evaluate the therapeutic effects of adaptable, non-invasive cortical modulation using rTMS on NOAB symptoms.

### Study objectives

The primary objective of this study is to examine the effects of rTMS in women with MS by investigating the restoration of functional connectivity in the brain, leading to improvements in urinary frequency, urgency, and incontinence.

The secondary objective is to enhance our understanding of the neural contributions to NOAB in MS, aiding in patient phenotyping and identifying potential urological and neurological indicators of response to rTMS. These objectives are achieved by evaluating three specific outcomes: (1) analyzing brain activation and connectivity using BOLD signals and FC of the targeted ROIs after rTMS sessions; (2) assessing voiding efficiency through UDS parameters and validated questionnaires that capture participant bladder symptoms and the presence of anxiety/depression following rTMS treatment; and (3) examining baseline UDS, clinical, and neuroimaging factors that may predict the response to rTMS treatment.

### Study population and recruitment

The study aims to recruit female patients with MS from our tertiary Neurourology clinic. Recruitment information is made readily available in our Urology and Neurourology clinical areas for interested patients. Eligible participants are adult women with clinically stable MS. Clinical stability is defined as the absence of exacerbation or worsening in the Expanded Disability Status Score (EDSS) score in the six months preceding the study entry. Patients with NLUTD symptoms persisting for at least three months are screened. See Table 1 for more detail of the inclusion/exclusion criteria. Individuals with active UTI will be treated and enrolled after negative urinalysis.

**Table 1:** Inclusion / exclusion criteria

Inclusion	Exclusion
• Adult women (≥ 18 years of age) • Clinically stable MS defined by an Expanded Disability Status Score (EDSS) ≤ 7.5 without exacerbation worsening in the preceding 6 months prior to study entry • Montreal Cognitive Assessment (MoCA) score > 10 • Neurogenic Lower Urinary Tract Dysfunction symptoms ≥ 3 months with NBSS total ≥ 15 • At least one bladder storage symptom (e.g., urinary frequency, urinary urgency, or nocturia with or without incontinence) indicated by OAB-AT ≥ 8	• Pregnant or planning on becoming pregnant or nursing • Bladder outlet obstruction • Baclofen or other intrathecal pump, pacemakers • History of seizure disorder (SZ), immediate family of SZ disorder • History of bipolar disorder • History of moderate to severe heart disease or unstable angina • History of autonomic dysreflexia (AD) • History of interstitial cystitis, or pelvic radiation • Intradetrusor BTX-A injections within 6 months prior to study participation • Active SNS or any other spinal stimulator • Indwelling urethral or suprapubic catheter

Exclusion criteria include vulnerable populations such as pregnant or planning to become pregnant, nursing, and incarcerated patients. The use of botulinum toxin for non-urologic conditions is permitted. Male participants are excluded due to potential complications arising from prostatic pathologies and anatomical variances.

### Power analysis

In this study, we define the response to treatment as a minimum improvement of 7.7 points on the Neurogenic Bladder Symptom Score (NBSS) Total score immediately after the application of active rTMS. This improvement represents the smallest real difference (SRD) of the total score, with a 90% confidence interval [41]. To evaluate the primary endpoint of significant improvement in the total NBSS score, we employ a two-sided, two-sample t-test for a 2-by-2 repeated measures design, with a significance level of 0.05. Based on these parameters, a sample size of *n* = 17 in the active treatment group and *n* = 9 in the sham group provides 80% power to detect a mean change of -4.4 in the total NBSS score between the two groups. The calculations consider a standard deviation of 5.9 at baseline, a standard deviation of 5.2 post-rTMS, and a correlation of 0.8 between measurement pairs, accounting for the expected non-responder rate. To ensure adequate enrollment, our goal is to include 26 patients, with an additional 3 patients (accounting for a potential 10% lost to follow-up), resulting in a total of 29 patients (active group: *n* = 19 and sham group: *n* = 10).

### Planned interventions

**Fig. 2:**
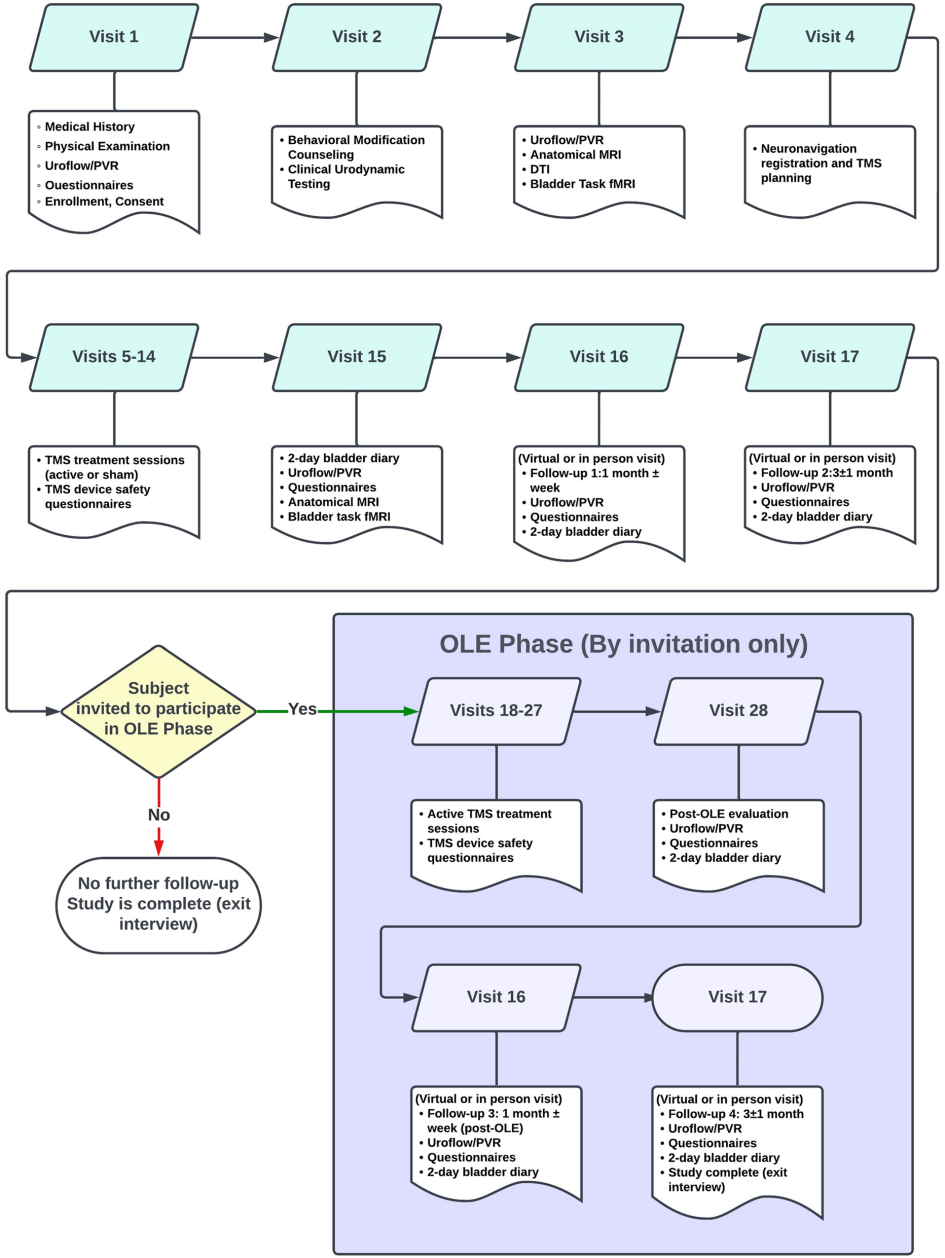
Participant visit flowchart

Participants attend a total of 17 visits in the initial phase of the study, and if invited to take part in the optional OLE phase, they attend a total of 30 visits. An overview of each visit can be found in Fig. 2.

#### Screening and recruitment (visits 1 and 2)

During the initial screening visit (visit 1), our research coordinator provides participants with detailed information about the study and addresses any questions or concerns they may have. Participants are given ample time to review all available study materials privately, including the Informed Consent Form. They may also choose to involve family members or seek a second opinion before making a decision about their participation. Upon providing consent, participants provide a comprehensive medical history and undergo a thorough physical examination. Visit 2 involves clinic urodynamics and counseling on behavioral modifications. Urine samples are collected for urinalysis and pregnancy testing. Participants have the option to combine visits 1 and 2.

Behavioral modification is considered the first-line treatment recommended by the American Urological Association/Society of Urodynamics, Female Pelvic Medicine & Urogenital Reconstruction (AUA/SUFU) for managing OAB and NOAB. After the screening and recruitment phase, we discuss behavioral modifications with the participants. The SUFU has provided clear guidelines for patients regarding fluid management, dietary changes, and techniques to alleviate urgency sensation and improve voiding.

#### Baseline scan (visit 3)

For the duration of this study, we utilize a 3-Tesla Siemens MAGNETOM Vida MRI scanner in our translational imaging core. To achieve higher resolution in both fMRI and diffusion tensor imaging (DTI), we employ a 64-channel head coil. This specific coil offers improved signal-to-noise ratio and improved spatial resolution compared to a standard 20-channel head coil. During this visit, we collect validated questionnaires and a two-day bladder diary. The validated questionnaires include assessments related to bladder symptoms such as the American Urological Association Symptom Score (AUASS) and the Incontinence Impact Questionnaire (IIQ-7). Additionally, we administer questionnaires addressing depression and anxiety, such as the Hospital Anxiety and Depression Scale (HADS). A comprehensive list of all assessments can be found in Table 2.

**Table 2:** Baseline / follow-up assessments (visits 1 and 2)

Assessment	Score/Measurement
• Expanded Disability Status Score (EDSS)	0—10 (Normal to Mortal)
• American Urological Assoc Symptom Score (AUASS)	0—35 (Mild to Severe)
• Neurogenic Bladder Symptom Score (NBSS)	0—74 (Normal to Max Symptoms)
• Urogenital Distress Inventory (UDI-6)	0—100 (Normal to Max Symptoms)
• Incontinence Impact Questionnaire (IIQ-7)	0—100 (Normal to Max Impact)
• Hospital Anxiety and Depression Scale (HADS)	0—42 (No Anxiety to Severe Anxiety)
• Hamilton Depression Rating Scale (HAM-D)	0—54 (No Depression to Severe Depression)
• Hamilton Anxiety Rating Scale (HAM-A)	0—56 (No Anxiety to Severe Anxiety)
• MRI Safety Questionnaire	Q&A
• 2-day Bladder Diary	Q&A
• Non-Instrumented Uroflow	mL/sec
• Post-Void Residual (PVR) Volume	cm^3^

### Pre-scan preparation

**Fig. 3:**
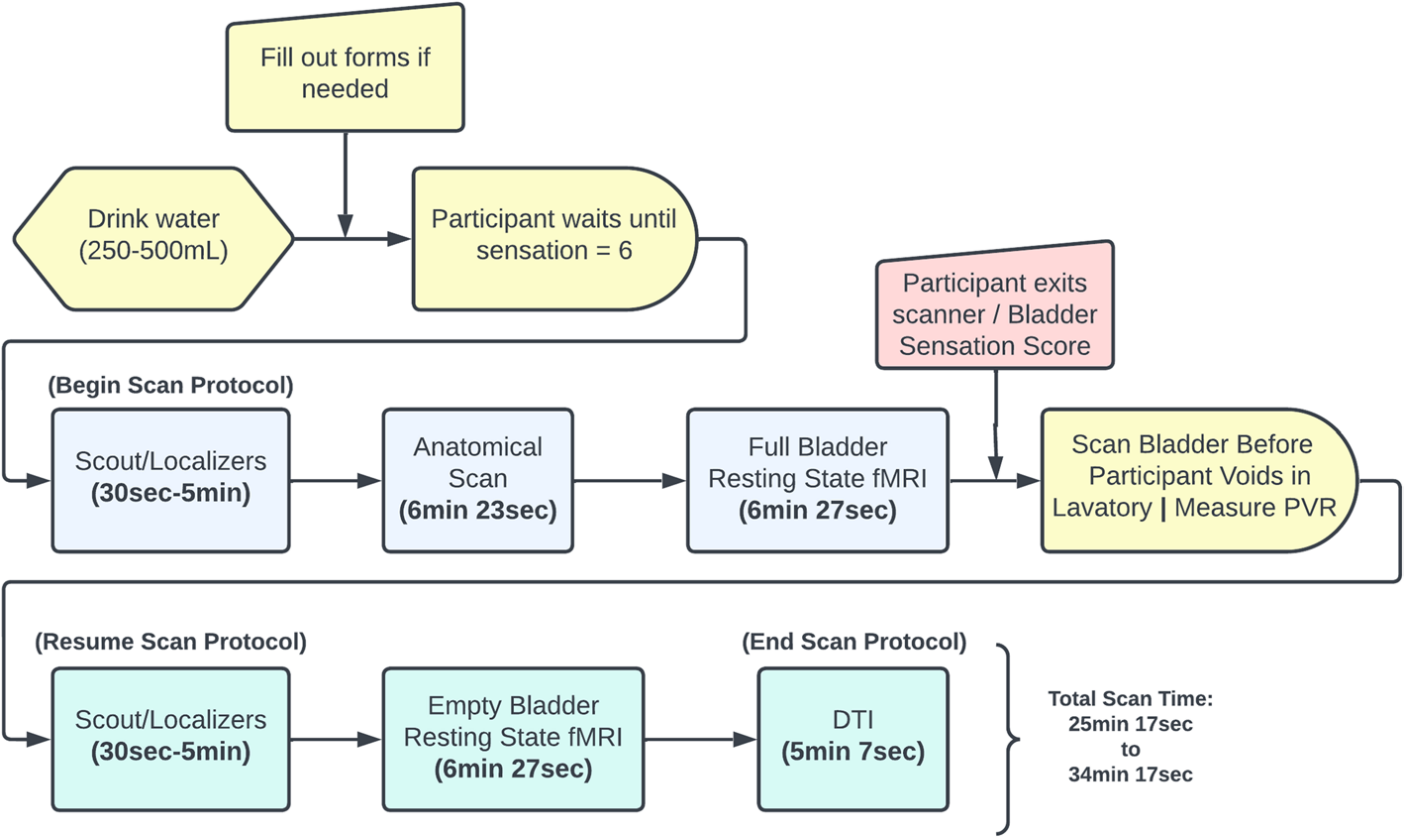
MRI scan protocol (Pre-TMS)

Prior to entering the MRI scanner, participants are instructed to consume a volume of water ranging from 250 to 500 mL. They then complete any necessary paperwork and wait until they perceive their bladder sensation to reach a level of 6 out of 10. At this point, the scan procedure is thoroughly explained to the participants, as depicted in Fig. 3. An MRI-compatible display also provides information to the participants about the ongoing scan. Technicians closely monitor the participants at all times, maintaining communication through an intercom system to ensure their understanding of each step and to address any discomfort they may experience. As a precautionary measure, patients are provided with a squeeze bulb that can be compressed to immediately stop the scanner and safely eject the gurney in case of an emergency.

### Scan procedure

Following localizer/scout programs, an isotropic T1-weighted GRAPPA anatomical scan is conducted with the following parameters: repetition time (TR) = 2000 ms, echo time (TE) = 2.51 ms, axial orientation, 1 mm voxel size. This is followed by the full bladder resting-state functional MRI (rs-fMRI) scan, utilizing an axial EPI BOLD sequence with the following parameters: TR = 2000 ms, TE = 30 ms, slice thickness of 3 mm, and 3 mm in-plane resolution. The scan procedure deviates from the norm as participants are asked to temporarily exit the scanner midway through the session to void in a nearby lavatory. During this time, non-instrumented uroflow and PVR volume are measured. Once the participants return to the scanner, a localizer/scout is performed to ensure they resume the same position as before. Subsequently, an empty bladder rs-fMRI scan is conducted with parameters analogous to the initial fMRI scan. Following this, an axial DTI scan with 64 directions is performed to facilitate the neuronavigation aspect of the rTMS therapy sessions (Fig. 3).

### Neuronavigation setup (visit 4)

Neuronavigation has become a widely used tool for planning interventions and neurosurgical procedures, offering improved precision in localizing anatomical and functional targets. By registering patients' brain scans (CT or MRI) in the neuronavigation system, a three-dimensional representation of the brain structure is generated, enabling surgeons to accurately identify tumor locations and other surgical intervention targets [42]. Neuronavigation is not limited to surgical applications but is increasingly utilized in trials requiring precise localization of specific brain structures, particularly in studies involving neuromodulation techniques like rTMS [43, 44]. Compared to conventional methods such as the 10–20 electroencephalography (EEG) system or distance estimation, neuronavigation provides superior precision in localizing areas for modulation due to the unique anatomical structures of each patient [45].

Recent studies in TMS coil positioning methods suggest that MRI-guided neuronavigation is preferable to the 10–20 EEG-based method of target determination due to individual asymmetries and other systematic differences [46, 47]. In addition, neuronavigation enables accurate identification of brain regions using the standardized Montreal Neurological Institute (MNI) coordinates, a commonly used coordinate system for MRI images, further facilitating the process of identifying target areas for neuromodulation [48]. By utilizing structural and functional MRI data for each individual, neuronavigation allows us to register the ROIs associated with the SMA, Iateral and medial pre-frontal cortex (lPFC/mPFC) activated during urges in each participant, enabling personalized neuromodulation based on their specific brain activity. It is worth noting that while some studies suggest a decrease in activation in the left IPFC following bladder treatment, its laterality varies in patients with OAB [49, 50]. The laterality of IPFC/mPFC neuromodulation is also individualized, targeting the side with higher activation of the anterior cingulate cortex (ACC)/Insula/SMA during urgency, determined using fMRI and neuronavigation.

During this visit, patients return to the clinic to have their rTMS ROIs selected and mapped. This process is identical for both the treatment and control groups. The anatomical and functional MRI scans of each participant, specifically capturing brain activation during "strong urgency," load into the neuronavigation system individually. The ROIs to be modulated locate using their MNI coordinates and confirmed based on their activation during "strong urgency." The corresponding positions of the rTMS probe are determined using tracking of the BrainSight system. The treatment group receives the active rTMS probe, while the control group receives the sham rTMS probe. The sham rTMS treatment is indistinguishable in appearance and sound from the active treatment sessions.

### Intervention: repetitive transcranial magnetic stimulation (rTMS)

rTMS is a non-invasive technique that can modify brain activity by applying a coil emitting rapid magnetic pulses to the scalp. This method allows for effective modulation of cortical neurons without the need for anesthesia or significant side effects. rTMS is commonly used in brain mapping [20, 21], adjusting cortical excitability, and treating conditions like depression, migraines, and obsessive–compulsive disorders [22–24]. The delivery of rTMS can be continuous with a low frequency (1 Hz) to inhibit brain activity or in bursts with a high frequency (5–10 Hz) to enhance it [51]. Through the modulation of cortical excitability, rTMS can induce lasting changes in neural connections within the brain [51, 52]. Previous studies, although limited in size and lacking a placebo control, have demonstrated the feasibility and effectiveness of using rTMS to specifically inhibit the SMA in individuals with MS and Parkinson's disease. These findings suggest the potential benefits of rTMS in improving NLUTD [25, 26].

For the rTMS therapy sessions, we utilize the MagStim Rapid 2 system (Fig. 3), which includes a figure-8-shaped magnetic coil. The coil is positioned on the scalp over the targeted area of the brain. It is connected to a stimulator that delivers a series of rapid electrical pulses, generating a rapidly changing magnetic field around the coil. To precisely target the activation centers for each patient, we employ the Brainsight TMS neuronavigation system in combination with anatomical MR images, fMRI analysis, and diffusion tractography. This integrated approach allows us to create a 3D map of each participant's brain activation and functional connectivity. By identifying specific areas of activation or lack thereof, we can tailor our modulation protocol to the corresponding brain regions.

### rTMS treatment setup (visits 5 – 14)

Over the course of two weeks on weekdays (visit 5–14), patients undergo a total of 10 rTMS treatment sessions in the clinic, receiving either active or sham stimulation. The treatment protocol begins with continuous inhibitory low-frequency rTMS (LF-rTMS) at 1 Hz for 20 min (1,200 pulses). This stimulation is applied at the midpoint between the identified MNI locations of the pelvic-SMA (right and left), determined using fMRI activation patterns and neuronavigation. Following a two-minute interval after completing the LF-rTMS, excitatory high-frequency rTMS (HF-rTMS) at 10 Hz is administered to the mPFC/lPFC, targeting the MNI locus identified during fMRI and neuronavigation. The HF-rTMS protocol consists of twenty 10-s trains at 10 Hz (10 pulses per second), with a 50-s pause between each train, totaling 20 min of HF-rTMS stimulation (2,000 pulses in total). The combined LF-rTMS and HF-rTMS procedure for the study protocol takes a total of 40 min, including a two-minute interval between the LF- and HF-rTMS sessions, delivering a total of 3,200 pulses. During each treatment session, patients complete a set of questionnaires before and after the stimulation to monitor for any potential complications related to rTMS. Additionally, they provide feedback on their perception of the type of treatment received after each session.

### Post-treatment and follow-ups (visits 15–17)

Within one week after the final active/sham rTMS treatment session, patients return to the clinic (visit 15) for follow-up assessments. During this visit, uroflow and post-void residual (PVR) volume measurements are taken; validated questionnaires and a two-day bladder diary are collected as well. Participants also undergo a repeat MRI protocol, excluding the DTI scan, replicating the imaging session from visit 3. This secondary imaging session allows for the analysis of fMRI images and a comparison to the subject's baseline fMRI images to identify any changes in activation patterns.

At the first follow-up, scheduled at 1 month ± 1 week after the rTMS treatment (visit 16), uroflow, PVR, and validated questionnaires are measured and collected. During this visit, patients are provided with information about the optional OLE component of the study, along with details about the assessments involved.

The second follow-up (visit 17) takes place at 3 months ± 1 month after the rTMS treatment. Patients who decline participation in the OLE phase undergo an exit interview. Those who choose to participate in the optional OLE phase are re-consented and receive 10 active rTMS treatment sessions in the clinic for two weeks on weekdays, irrespective of their original randomization assignment.

### Open-label extension (OLE) phase (visits 18–27)

During visits 18–26, patients in the OLE phase undergo a total of 10 active rTMS treatment sessions in the clinic for two weeks on weekdays. Each treatment session lasts 40 min. As in previous phases, patients complete questionnaires before and after each session to assess any potential complications associated with rTMS. Furthermore, they provide feedback on their perception of the type of treatment received after each session. The OLE phase allows for a longer-term evaluation of rTMS treatment effectiveness while gathering additional data to further elucidate the tolerability and safety profiles of this therapeutic modality.

### Randomization and blinding

Participants are randomized into two groups: Group 1, consisting of the active rTMS (treatment group, *n* = 19), and Group 2, comprising the sham rTMS (control group, *n* = 10). The randomization process employs a blocked randomization technique with a block size of 6 and an allocation ratio of 2:1 to assign participants to either the active or sham rTMS group. The study coordinator receives a computer-generated randomization list generated using STATA version 16. Blinding is maintained throughout the entire study, with the PI, co-PI, research personnel responsible for data analysis, and participants unaware of the group assignment.

### Study outcome measures

The primary outcome measures of this study involve assessing the improvement in patients' NOAB symptoms using subjective and objective clinical data. We collect validated questionnaires, bladder diary entries, and measurements of uroflow and PVR immediately, 1 month, and 3 months after treatment in both the treatment (active) and control (sham) groups.

Secondary outcome measures focus on the analysis of BOLD signal activation and functional connectivity (FC) patterns during resting state and periods of "strong desire to void" in predefined ROIs. We conduct these analyses at baseline and immediately following treatment in both the active and sham groups. We examine task-based FC, particularly during episodes of urinary urgency, to gain insights into underlying brain function and network behavior. This assessment is particularly relevant for individuals with MS as lesion location may not directly affect bladder function regions but can impact communication between these regions.

### Clinical data analysis

Baseline characteristics of the patients are summarized according to their respective treatment groups. A paired t-test or, alternatively, a Wilcoxon signed-rank test (for nonparametric data) is employed to compare subjective (validated questionnaires) and objective (bladder diary, non-instrumented uroflow variables, and PVR measured using a bladder scanner) variables between the baseline and post-treatment time points within each group. Two-way Analysis of Variance (ANOVA) or mixed-effect model for repeated measures (MMRM) analysis is conducted using all available longitudinal data. A delayed start analysis, utilizing an MMRM approach and incorporating data from both the early start and OLE periods, is also performed. All statistical analyses are carried out using STATA version 16 statistical software, with a significance level of *p* < 0.05 (two-tailed) applied to all tests.

### Neuroimaging data analysis

#### BOLD analysis

Pre-processing of structural and functional images is conducted using FSL software. This involves co-registering and motion correcting the images, while patients with excessive motion are excluded from the analysis. Voxel activation is identified during the "strong urgency" period. First and second-level (group) analyses are performed using CONN (Version 22a), a functional connectivity toolbox for MATLAB (Version 2021b), which utilizes the statistical parametric mapping (SPM12) MATLAB toolbox. Significantly differentially activated voxels are identified during "strong urgency" using a generalized linear model (GLM). Group analysis is conducted by transforming the data into Talairach space, and significant voxels are identified using a student's t-test. Comparisons between baseline and post-treatment rTMS scans are made for each group, examining BOLD signals derived from patient fMRI scans to assess changes in ROI activation.

#### FC analysis

Functional connectivity (FC) analysis involves utilizing CONN to generate separate FC matrices from BOLD data that has been aligned to a shared space (Talairach space). FC refers to the temporal correlation between distant neurophysiological events, as evaluated through their respective BOLD signal time courses. CONN software is employed for conducting FC analysis. A region-based connectivity analysis is carried out before and after treatment, utilizing regions of interest defined in a brain atlas accessible within the toolbox. FC is quantified using T-values, considering a two-sided, FDR-corrected *p*-value threshold of less than 0.05. Additionally, CONN software is used to compute fractional anisotropy (FA) and mean diffusivity (MD) images, and to align individual maps with the ICBM WMPM white matter atlas. This facilitates automated processing and group-level analysis of specific white matter tracts of interest, including their FA and MD values. Lastly, machine learning algorithms implemented in the R language package caret are employed to identify the most statistically significant differences in both functional and anatomical connectivity between groups.

### Data and safety monitoring

All members of the research team maintain up-to-date training in safeguarding human research participants and strictly adhere to HIPAA regulations. Moreover, we employ stringent data security and data encryption protocols to protect subject confidentiality and minimize data breach risks.

Safety oversight is conducted by a Data Safety Monitoring Committee comprised of an independent team of specialists, including a Neurologist/Neurosurgeon, Urologist, and Physiatrist who possess expertise in MS, NLUTD, and neuromodulation cases/trials. These committee members do not directly participate in the study's implementation but possess sufficient knowledge about the project and perform regular reviews of study data bi-annually. Their primary focus is evaluating patient safety, privacy, confidentiality, and treatment efficacy. At the conclusion of each meeting, the committee provides a comprehensive written report containing detailed feedback and recommendations.

### Hard stops for individual participants

In the event that a participant experiences any discomfort during the interventions (fMRI, neuronavigation, rTMS), we promptly evaluate and address any symptoms. We consult our safety monitoring team and IRB and proceed based on their recommendations. Participants with a history of seizures are excluded from the study. However, if such an event occurs despite appropriate clinical care, the participant is discontinued from further participation. The safety monitoring committee reviews reports of any discomfort or unintended/adverse side effects. If the IRB or safety monitoring committee recommends it, the study may be halted at any point.

## Discussion

The primary strength of this study is that it explores a novel therapeutic option for MS with NOAB and provides insights into brain mechanisms during "strong urgency," a urological process that has barely been studied under a brain function paradigm, especially in NLUTD patients. Nonetheless, limitations do exist.

Since MS is a heterogeneous disease with dynamic manifestations, patients within this trial represent only a subset of a larger and more complex group that may have inter- and intra-subject variability. Our inclusion criteria are restricted to only MS patients with predominantly NOAB and > 6 months with clinically stable MS. While it is true that lesion location and burden is stochastic, this approach will homogenize the group and will address the potential of disease variation in NLUTD and rTMS. Another concern that can arise during this study is that neuromodulation requires the targeted neural network to be functional, which can be challenging in MS as there are variable degrees of white matter damage with varying lesion locations (brain and spinal cord). Manifestation and characterization of spinal lesions in MS is extremely difficult and variable and clinical symptoms are not associated with radiologic findings [53, 54]. It would be nearly impossible to exclude all subjects with spine lesions as many MS patients have cord pathology which is often missed in standard clinical spinal MRI [55]. Despite such limitations, with recent advances in disease modifying treatments, MS research has shifted focus to neuromodulation and brain restoration [56].

Targeted neuromodulation of the ROIs in circuits 1 and 2 that feed into PAG will take us one step closer to understanding the fundamental mechanisms and causal relationships between these supraspinal circuits and bladder symptoms. Our study will, for the first time, assess a mechanistic neuromodulatory approach in women with MS and NOAB to restore the function of the pathways within circuits 1 and 2 to a level of activation observed in normal controls.

## Data Availability

The data generated during this study will not be made publicly available to protect the privacy of the participants, but can be provided by the corresponding author on reasonable request and based on approval from relevant authorities. Overall trial results will be communicated through peer-reviewed publications and outcomes are to be posted on ClinicalTrials.gov.

## Abbreviations

AUA: American Urological Association
AUASS: American Urological Association Symptom Score
ACC: Anterior Cingulate Cortex
ACs: Anticholinergics
ANOVA: Analysis of Variance
BOLD: Blood Oxygen Level Dependent
BTX-A: Botulinum Toxin Type A
DTI: Diffusion Tensor Imaging
EDSS: Expanded Disability Status Scale
EEG: Electroencephalography
FC: Functional Connectivity
FA: Fractional Anisotropy
FDA: U.S. Food and Drug Administration
fMRI: Functional Magnetic Resonance Imaging
GRAPPA: Generalized Auto-calibrating Partial Parallel Acquisition
HF-rTMS: High-Frequency Repetitive Transcranial Magnetic Stimulation
IIQ-7: Incontinence Impact Questionnaire-7
LF-rTMS: Low-Frequency Repetitive Transcranial Magnetic Stimulation
lPFC: Lateral Prefrontal Cortex
LUT: Lower Urinary Tract
MD: Mean Diffusivity
MNI: Montreal Neurological Institute
MMRM: Mixed Model for Repeated Measures
MRI: Magnetic Resonance Imaging
mPFC: Medial Prefrontal Cortex
NBSS: Neurogenic Bowel Dysfunction Score
NLUTD: Neurogenic Lower Urinary Tract Dysfunction
NOAB: Neurogenic Overactive Bladder
OAB: Overactive Bladder
PVR: Post-Void Residual
PTNS: Percutaneous Tibial Nerve Stimulation
ROI: Region of Interest
Rs-fMRI: Resting-state Functional Magnetic Resonance Imaging
rTMS: Repetitive Transcranial Magnetic Stimulation
SZ: Seizure Disorder
SNS: Sacral Nerve Stimulation
SRD: Stress-related Disorders
SUFU: Society of Urodynamics, Female Pelvic Medicine & Urogenital Reconstruction
TMS: Transcranial Magnetic Stimulation
TE: Echo Time
TR: Repetition Time
UDS: Urodynamic Study
UUI: Urgency Urinary Incontinence
UTI: Urinary Tract Infection
